# Impact of the COVID-19 pandemic on quality of life and mental health in children and adolescents in Germany

**DOI:** 10.1007/s00787-021-01726-5

**Published:** 2021-01-25

**Authors:** Ulrike Ravens-Sieberer, Anne Kaman, Michael Erhart, Janine Devine, Robert Schlack, Christiane Otto

**Affiliations:** 1 Department of Child and Adolescent Psychiatry, Psychotherapy, and Psychosomatics, University Medical Center Hamburg-Eppendorf, Hamburg, Germany; 2 Alice Salomon University of Applied Science, Berlin, Germany; 3 Apollon University of Applied Science of Healthcare Economy, Bremen, Germany; 4 Argora Clinic, Psychosomatic Clinic and Outpatient Center, Berlin, Germany; 5 Department of Epidemiology and Health Monitoring, Robert Koch Institute, Berlin, Germany

**Keywords:** COVID-19, Mental health, Quality of life, Anxiety, Depression, Children and adolescents

## Abstract

The COVID-19 pandemic has caused unprecedented changes in the lives of 1.6 billion children and adolescents. First non-representative studies from China, India, Brazil, the US, Spain, Italy, and Germany pointed to a negative mental health impact. The current study is the first nationwide representative study to investigate the impact of the COVID-19 pandemic on health-related quality of life (HRQoL) and mental health of children and adolescents in Germany from the perspective of children themselves. A representative online survey was conducted among *n* = 1586 families with 7- to 17-year-old children and adolescents between May 26 and June 10. The survey included internationally established and validated instruments for measuring HRQoL (KIDSCREEN-10), mental health problems (SDQ), anxiety (SCARED), and depression (CES-DC). Results were compared with data from the nationwide, longitudinal, representative BELLA cohort study (*n* = 1556) conducted in Germany before the pandemic. Two-thirds of the children and adolescents reported being highly burdened by the COVID-19 pandemic. They experienced significantly lower HRQoL (40.2% vs. 15.3%), more mental health problems (17.8% vs. 9.9%) and higher anxiety levels (24.1% vs. 14.9%) than before the pandemic. Children with low socioeconomic status, migration background and limited living space were affected significantly more. Health promotion and prevention strategies need to be implemented to maintain children’s and adolescents’ mental health, improve their HRQoL, and mitigate the burden caused by COVID-19, particularly for children who are most at risk.

**Supplementary Information:**

The online version contains supplementary material available at 10.1007/s00787-021-01726-5.

## Introduction

The Coronavirus Disease 2019 (COVID-19) pandemic has led to rapid, unprecedented changes to the lives of billions of children and adolescents. Faced with countless deaths and hundreds of thousands of people worldwide being infected, most countries have implemented massive preventive measures. The prevalence of COVID-19 in children is low (between 0.8% and 3.3%) and most children only display mild physical symptoms or are asymptomatic [1–4]. However, while COVID-19 may not be as severe and deadly in children as it is in adults, its impact on their health-related quality of life (HRQoL) and mental health is not yet understood sufficiently well. Children and adolescents face massive changes in their daily lives, including school closures, home confinement, and social distancing rules, which can burden them substantially [5–7]. Furthermore, violence against children is reported to have increased under home confinement leaving children at risk of abuse and trauma [8]. Particularly children with low socioeconomic status and preexisting mental health problems may be exposed to cumulative risks. This assumption is based on a solid body of previous research demonstrating that a low socioeconomic status of children [9–11], low parental education and migration status [12, 13] are risk factors for mental health problems among children. Also when experiencing home confinement, it can be assumed that children living in small apartments are more stressed—concluding from the literature indicating that limited living space can affect mental health [14]. During the pandemic children also receive substantially less paediatric healthcare if outpatient daycare centers are closed, resulting in some children’s diseases remaining untreated [15].

Childhood and adolescence involve numerous biopsychosocial changes and challenges, including gaining independence from parents, exploring various domains of identity, and coping with difficulties in everyday life and at school [16]. Adolescence is a sensitive period for social development with an increased need for social interactions [7]. Coping with the current situation and complying with the current restrictions on top of this can be especially difficult for children and adolescents since these circumstances can be experienced as being incongruent with their developmental tasks. The challenges and consequences of COVID-19 might therefore have a tremendous impact on their HRQoL and mental health.

Currently, there is a growing interest in research on the mental health of children and adolescents during the COVID-19 pandemic. First non-representative studies from China, where COVID-19 spread earliest and initially affected most people, reported increasing levels of stress, anxiety and depression. Jiao et al. [17] report that one-third of 3- to 18-year-old children and adolescents were clingy, inattentive, irritable and worried. Xie et al. [18] found that 23% of 2nd- to 6th-grade children had depressive symptoms and 19% had anxiety symptoms during the pandemic, while Zhou et al. [19] report that 44% of 12- to 18-year-olds displayed depressive symptoms, 37% showed anxiety, and 31% had both types of symptoms. High levels of depressive and anxiety symptoms were recently replicated by Duan et al. [20]. Two non-representative studies from India with children and adolescents aged 5–18 years [21, 22] and one study from Brazil with children and adolescents from 6 to 12 years [23] underline the negative impact of the pandemic on the mental health of children. They found that children and adolescents experienced severe psychological distress such as worries, helplessness, anxiety and fear. Moreover, recent nationwide studies from the US reported worsening psychological well-being and behavioural health of children and adolescents compared to the time before the pandemic [24, 25]. Further, two European non-representative studies from Italy and Spain found that mental health problems such as conduct problems, irritability and loneliness in children and adolescents increased during the COVID-19 lockdown [26, 27]. One non-representative survey among parents of German children and adolescents [28] and one qualitative German study [29] further found that children and adolescents are often worried about the COVID-19 pandemic and the associated changes.

Under normal circumstances, the worldwide prevalence of mental disorders is 13.4% as reported by a meta-analysis [30]. The above-mentioned studies thus indicate a significant increase in mental health problems in children during the pandemic, which may lead to manifest disorders over time. There is still a lack of knowledge about how this prevalence has or will increase while the pandemic continues.

This representative COPSY study (impact of **CO**VID-19 on **psy**chological health) aims to explore the HRQoL and mental health of children and adolescents aged 7–17 years during the COVID-19 pandemic and to compare it to pre-pandemic data of the nationwide representative study of the **be**haviour and we**l**lbeing of chi**l**dren and **a**dolescents in Germany (BELLA study) [31, 32].

Our main hypotheses were:

1. Children’s and adolescents’ HRQoL and mental health are impaired during the COVID-19 pandemic. Specifically, we expected that children and adolescents feel burdened by the pandemic, show a decrease in HRQoL, an increase in mental health problems, higher levels of anxiety, depression and more psychosomatic symptoms during the pandemic compared to before the pandemic.
2. Some children and adolescents are particularly impaired by the COVID-19 pandemic. We assumed that children and adolescents with low socioeconomic status, migration background and limited living space are affected significantly more.

We aim to identify children’s and adolescents’ needs during the pandemic and offer guidance to policymakers, paediatric professionals and parents for safeguarding the mental health of children.

## Methods

### Study design and sample

The nationwide, population-based COPSY study was conducted in Germany between May 26 and June 10, 2020, while the country was under a partial lockdown, with schools, leisure facilities and most stores closed and with preventive distancing measures in place. The COPSY study was approved by the Local Psychological Ethics Committee (LPEK-0151) and the Commissioner for Data Protection of the University of Hamburg. Overall, *n* = 3597 families with children and adolescents aged 7–17 years were invited to participate in the survey. They were contacted, informed about the study and asked for their informed consent. A total of *n* = 1647 families consented and completed the online survey via the survey software EFS Survey from Questback. The participation rate was 45.8% (calculated according to a formula by the American Association for Public Opinion Research, AAPOR [33]). After data cleaning (*n* = 61 participants were excluded due to implausible data), the final sample included *n* = 1586 families. Self-reports of children and adolescents were gathered if aged at least 11 years (*n* = 1040, 11–17 years old, one child per family). Parent proxy-reports were gathered of 11- to 17-year-olds who provided self-reports (*n* = 1040) and of younger children aged 7–10 years (*n* = 546). The weighted data of the final study sample matched the sociodemographic characteristics of the German population (based on the 2018 microcensus; the individual weights ranged from 0.2 to 3.8).

The COPSY study design and methodology is similar to the nationwide, longitudinal, representative BELLA study, though samples differed on the individual level across both studies. The BELLA study is the mental health module within the German Health Interview and Examination Survey for Children and Adolescents (KiGGS) [34] which administered established and validated questionnaires on HRQoL and mental health to children, adolescents and parents. Details of the BELLA study are described elsewhere [31, 32]. Extensive data from the BELLA study conducted in Germany in 2017 (*n* = 1556) were used to compare the data from the COPSY study with those of population-based reference samples surveyed prior to the COVID-19 pandemic. Due to the availability of data in different questionnaires, corresponding subsamples of the BELLA study were used for comparison.

### Measures

#### Sociodemographic variables

Children and adolescents aged 11–17 years responded to the self-report version of the online survey, parents of children aged 7–17 years answered the parent proxy version of the online survey. For sociodemographic information, the child and adolescent survey included questions on age and gender, the parent proxy survey included questions on age, gender, marital status, occupational status, parental education and migration background.

#### COVID-19 burden

To explore the burden of the pandemic, both versions of the online survey (self- and parent proxy-reports) included self-developed pandemic-focused items (drawing from our expertise in developing the KIDSCREEN measures). We asked for the perceived overall burden of the COVID-19 pandemic and for the burden caused by social distancing and school closures as well as effects of the pandemic situation on family climate.

#### HRQoL and mental health

To assess the impact of the pandemic on HRQoL and mental health, internationally established, comparable and validated instruments following recommendations by the International Consortium for Health Outcomes Measurement (ICHOM) [35] were used. Self- and parent proxy-reports of the survey included the established KIDSCREEN-10 Index [36], the scale on generalized anxiety from the German version of the Screen for Child Anxiety Related Disorders (SCARED) [37] and selected items from the German version of the Center for Epidemiological Studies Depression Scale (CES-DC) [38]. The Strengths and Difficulties Questionnaire (SDQ) [39] on mental health problems in children and adolescents was only administered in the parent proxy survey of the COPSY study. These measures were not only used in the COPSY study but also in the BELLA study so that a comparison of HRQoL and mental health before and during the pandemic was possible.

The KIDSCREEN-10 Index provides a global HRQoL score covering the physical, psychological, and social facets of HRQoL [36]. Its ten items (e.g., “Have you felt full of energy?”) were presented with 5-point response scales (0 = “never” to 4 = “always” or 0 = “not at all” to 4 = “extremely”). The mean score ranges from 0 to 4. Scores one standard deviation below the population mean (before the pandemic) were categorized as “low” HRQoL. The Strengths and Difficulties Questionnaire (SDQ) [39] assesses mental health with four problem scales: emotional symptoms, conduct problems, hyperactivity, and peer problems. Each problem scale consists of five items presented with three response options (0 = “not true” to 2 = “certainly true”). According to published cut-offs, we categorized participants based on the sum scores into groups according to their mental health (noticeable/abnormal, borderline and normal) [40]. The German version of the Screen for Child Anxiety Related Disorders (SCARED) [37] includes nine items on symptoms of generalized anxiety (e.g., “I worry about what is going to happen in the future”) which are presented with a 3-point response scale (0 = “not true or hardly ever true” to 2 = “very true or often true”). These 9 items are gathered in a sum score with higher scores indicating more severe symptoms of generalized anxiety. The scale score was used to categorize participants into two groups (those with versus those without generalized anxiety) based on the provided cutoff by Birmaher et al. [37]. Seven items of the Geman version of the Center for Epidemiological Studies Depression Scale (CES-DC) [38] (e.g., “I felt sad”) were administered and presented with a 4-point response scale (0 = “not at all” to 3 = “a lot”). A mean score gathering all items was calculated with higher scores indicating more severe depressive symptoms. In the COPSY study, the internal consistency was good for the analyzed self-reported KIDSCREEN-10 Index, for the parent-reported SDQ total score, and for self-reported generalized anxiety and depression scores (α = 0.82, 0.84, 0.89, 0.84). In the sample used from the BELLA study, the internal consistency for the scales was mainly comparable (in the order presented above: α = 0.80, 0.82, 0.83, 0.79).

Finally, psychosomatic complaints were assessed in self- and parent proxy-reports using the HBSC symptom checklist [41]. The HBSC symptom checklist assesses how often children and adolescents experienced eight different psychosomatic complaints (e.g., headaches, sleeping problems, irritability) during the last week. Items were offered with a 5-point response scale (0 = “not at all” to 4 “daily”). The BELLA study did not provide comparison data for this scale.

### Data analysis

To investigate the perceived burden of the pandemic, the pandemic-specific items were examined via descriptive statistics (frequencies, means and standard deviations). To evaluate differences in HRQoL and mental health before and during the pandemic, cross-sectional data from the pre-pandemic BELLA study (control group) and the pandemic COPSY study (index group) were pooled; depending on data availability, two different BELLA subsamples were used. Prior to multivariate regression analyses with pooled data, bivariate analyses were conducted (cross-tabulation, chi-square tests, t-tests and ANOVAs). The regression models with pooled data were controlled for age, gender, age*gender, parental education and migration status. We considered a *p* value ≤ 0.05 as an indicator for significant differences or effects.

Prior to conducting data analyses, a power analysis was conducted. Sample size was calculated to test for statistical significance with *p* (alpha) < 0.05 and a power of *p* = 0.8 for moderate effect (*d* = 0.5) between two groups in a particular age (7–10; 11–13; 14–17) and gender (females vs. males) group. This power calculation leads to *n* = 612 respondents at minimum. The power calculation was conducted with the G-Power 3.1 software.

As the BELLA study did not provide comparative data on psychosomatic complaints, the responses to the HBSC symptom checklist were presented using descriptive statistics only.

To examine which children are at higher risk of being particularly impaired by the pandemic, first age and gender differences were explored in detail. Then a high-risk analysis was conducted. Based on a-priori theoretical considerations, children with a certain sociodemographic and psychosocial profile were considered as being at higher risk and the resulting group was examined for impairments in the main study outcomes. Effects were described as mean differences and Cohens d-effect size measures.

All analyses were performed using SPSS version 26.

## Results

### Sociodemographics

Data from *n* = 1586 families with children aged 7–17 years (unweighted data: *M*_*age*_ = 12.25, *SD*_*age*_ = 3.30, *n* = 793 [50.0%] female) were analysed (self- and parent proxy-reports). The majority of the children and adolescents had no migration background [*n* = 1332 (84.0%)]. Most of their parents had a medium level of education [*n* = 884 (55.7%)], were married [*n* = 1097 (69.2%)] and were employed full-time [*n* = 820 (51.7%)]. Further details on the sociodemographic characteristics of the COPSY sample are presented in Table 1. The sociodemographic characteristics of the COPSY and BELLA subsamples used for the pooled regression analyses on HRQoL and mental health of children and adolescents before and during the COVID-19 pandemic are depicted in Supplementary Tables 1 and 2.

**Table 1:** Sociodemographic characteristics of the COPSY sample

	Parents of children aged 7–17 years (*n* = 1586)		Children and adolescents aged 11–17 years (*n* = 1040)	
	*n* (%)	*M (SD)*	*n* (%)	*M (SD)*
Age of the child		12.25 (3.30)		14.33 (1.86)
7–10 years	546 (34.4)		–	
11–13 years	351 (22.1)		351 (33.8)	
14–17 years	689 (43.4)		689 (66.3)	
Gender of the child				
Male	791 (49.9)		508 (48.8)	
Female	793 (50.0)		531 (51.1)	
Diverse	1 (0.1)		1 (0.1)	
No information	1 (0.1)		–	
Age of the parent		43.99 (7.36)		46.28 (6.74)
Migration background				
No	1332 (84.0)		879 (84.5)	
Yes	254 (16.0)		161 (15.5)	
Parental education				
Low	288 (18.2)		192 (18.5)	
Medium	884 (55.7)		548 (52.7)	
High	383 (24.1)		277 (26.6)	
No information	31 (2.0)		23 (2.2)	
Marital status				
Unmarried	140 (8.8)		87 (8.4)	
Married	1097 (69.2)		717 (68.9)	
In a relationship	216 (13.6)		125 (12.0)	
In a registered partnership	13 (0.8)		8 (0.8)	
Divorced	108 (6.8)		92 (8.8)	
Widowed	12 (0.8)		11 (1.1)	
Occupational status				
Full-time employed	820 (51.7)		561 (53.9)	
Part-time employed	453 (28.6)		286 (27.5)	
Self-employed	67 (4.2)		49 (4.7)	
Other employment	32 (2.0)		22 (2.1)	
Housewife/househusband	109 (6.9)		61 (5.9)	
Retiree/pensioner	34 (2.1)		27 (2.6)	
On parental leave	29 (1.8)		7 (0.7)	
Unemployed	42 (2.6)		27 (2.6)	
COVID-19 infection				
A family member was infected	60 (3.8)		35 (3.4)	
A relative died of COVID-19	29 (1.8)		22 (2.1)	

Unweighted data

*M* mean, *SD* standard deviation

### Perceived burden of the pandemic

Two-thirds [weighted data: *n* = 735 (70.7%)] of the children and adolescents (aged 11–17 years) stated that they felt burdened by the COVID-19 pandemic. More than half of the children and adolescents found homeschooling and learning to be more difficult than before the pandemic [*n* = 670 (64.4%)], the majority reported fewer social contacts during the pandemic [*n* = 861 (82.8%)], and nearly two-fifth of the children and adolescents stated that their relationships with their friends had been impaired [*n* = 408 (39.3%)]. About a fourth of the children and adolescents reported that arguments had increased in the family [*n* = 287 (27.6%)]. Using parent proxy-reported data (parents of 7- to 17-year-olds), about a third of the parents stated that disputes escalated more often [*n* = 508 (32.0%)].

### HRQoL before vs. during the pandemic

Before the pandemic, 15.3% (*n* = 146; based on weighted data of the BELLA study) of children and adolescents reported low HRQoL; during the pandemic, 40.2% of the children and adolescents reported low HRQoL (*n* = 418; based on weighted self-reported data of the COPSY study). An analysis stratified by gender revealed that a higher proportion of girls reported low HRQoL than their male peers both before and during the pandemic (Table 2). Younger children were affected significantly more than older ones; the percentage of children reporting low HRQoL rose from 7.7% to 41.3% in 11- to 13-year-old children and from 17.1% to 39.3% in 14- to 17-year-olds (*p* < 0.001).

**Table 2:** HRQoL in children and adolescents before vs. during the COVID-19 pandemic, stratified by gender (self-report, 11–17 years)

	Low HRQoL^a^	Normal/high HRQoL^a^
Boys		
Before pandemic (*n* = 492)	10.4%	89.6%
During pandemic (*n* = 524)	35.7%	64.3%
	*p*^b^ < .001	
Girls		
Before pandemic (*n* = 460)	20.4%	79.6%
During pandemic (*n* = 515)	44.7%	55.3%
	*p* < .001	
Boys and girls		
Before pandemic (*n* = 982)	15.3%	84.7%
During pandemic (*n* = 1039)	40.2%	59.8%
	*p* < .001	

^a^Groups low and normal/high HRQoL according to the KIDSCREEN, for details, see Methods

^b^*p* values resulting from *χ*^2^ test comparing the two groups of children and adolescents with low vs. normal/high HRQoL across the pre-pandemic BELLA study and the COPSY study during the pandemic

### Mental health before vs. during the pandemic

Based on parent proxy-reports, 7- to 17-year-old children and adolescents suffered from more mental health problems compared to before the pandemic. The prevalence of noticeable mental health problems was 9.9% (*n* = 153) before the pandemic and increased to 17.8% (*n* = 283) during the pandemic. This increase was significantly higher in 7- to 10-year-olds (from 7.4% to 26.8%) compared with 11- to 13-year-olds (from 12.8% to 14.5%) (*p* < 0.001).

Considerable rates for parent-reported hyperactivity [*n* = 233 (14.6%)], emotional problems [*n* = 210 (13.3%)], peer problems [*n* = 183 (11.5%)] and conduct problems [*n* = 159 (10.0%)] were found during the pandemic. In an analysis stratified by gender, different gender-specific patterns of mental health problems were found before and during the pandemic (Table 3).

**Table 3:** Mental health problems in children and adolescents before vs. during the COVID-19 pandemic, stratified by gender (parent-report, 7–17 years)

	Mental health problems (total)	Emotional symptoms	Conduct problems	Hyperactivity	Peer problems
Boys					
Before pandemic (*n* = 793)					
Normal^a^	81.3%	88.3%	84.5%	84.4%	88.9%
Borderline^a^	8.8%	4.3%	8.1%	5.4%	3.5%
Noticeable/abnormal^a^	9.8%	7.4%	7.4%	10.2%	7.6%
During pandemic (*n* = 816)					
Normal	66.2%	80.6%	77.5%	70.3%	76.2%
Borderline	14.1%	8.0%	10.9%	11.3%	10.3%
Noticeable/abnormal	19.7%	11.4%	11.6%	18.4%	13.5%
	*P*^*b*^ < .001	*p* = .007	*p* = .004	*p* < .001	*p* < .001
Girls					
Before pandemic (*n* = 760)					
Normal	83.4%	78.7%	89.5%	90.1%	88.3%
Borderline	6.7%	8.3%	4.9%	4.7%	4.3%
Noticeable/abnormal	9.9%	13.0%	5.7%	5.1%	7.4%
During pandemic (*n* = 768)					
Normal	73.3%	77.2%	84.4%	82.8%	80.4%
Borderline	10.8%	7.4%	7.3%	6.4%	10.1%
Noticeable/abnormal	15.9%	15.3%	8.3%	10.8%	9.5%
	*p* < .001	*p* = .198	*p* = .042	*p* < .001	*p* = .137
Boys and girls					
Before pandemic (*n* = 1553)					
Normal	82.4%	83.6%	86.9%	87.2%	88.6%
Borderline	7.8%	6.2%	6.5%	5.1%	3.9%
Noticeable/abnormal	9.9%	10.2%	6.6%	7.7%	7.5%
During pandemic (*n* = 1585)					
Normal	69.6%	79.0%	80.8%	76.4%	78.2%
Borderline	12.5%	7.7%	9.1%	8.9%	10.2%
Noticeable/abnormal	17.8%	13.3%	10.0%	14.6%	11.5%
	*p* < .001	*p* = .007	*p* < .001	*p* < .001	*p* < .001

^a^Groups due to mental health problems according to the SDQ, for details, see Methods

^b^*p*—values resulting from *χ*^2^—tests comparing groups normal and borderline (gathered into one group) vs. noticeable/abnormal according to the SDQ across the pre-pandemic BELLA study and the COPSY study during the pandemic

Based on self-reported data of 11- to 17-year-olds, the children and adolescents experienced higher levels of generalized anxiety during the COVID-19 pandemic [*n* = 251 (24.1%)] compared with before the pandemic [*n* = 198 (14.9%)].

The children and adolescents also self-reported depressive symptoms: 62.1% (*n* = 646) had trouble concentrating, 58.4% (*n* = 607) had little interest or joy in activities, and 33.7% (*n* = 351) felt sad. Surprisingly, no significant increase (*p* > 0.05) was found in the prevalence of depressive symptoms before vs. during the pandemic.

Linear regression analyses indicated significant differences between COPSY (during pandemic) and BELLA (before pandemic) data on almost all mental health outcomes (Table 4). Effects were small for parent-reported total mental health problems, hyperactivity and peer problems (Cohen’s *f*^*2*^ = 0.04, 0.03, 0.05), and negligible for parent-reported conduct problems and self-reported generalized anxiety (Cohen’s *f*^*2*^ = 0.01).

**Table 4:** Mental health impact of COVID-19 measures on children and adolescents

	Constant	Age	Female	Age*female	Education	Migration background	During vs. before pandemic	*Adjusted R*^*2*^
	B coeff (95% CI)	B coeff (95% CI)	B coeff (95% CI)	B coeff (95% CI)	B coeff (95% CI)	B coeff (95% CI)	B coeff (95% CI)	
HRQoL^a^	**51.74** (47.31;56.16)	0.08 (− 0.22;0.37)	**8.72 **(2.96;14.48)	**− 0.72 **(− 1.11;− 0.32)	− 0.00 (− 0.18;0.17)	− 1.03 (− 2.11;0.05)	− **6.51 **(− 7.28;− 5.74)	0.142
Mental health problems (total)^b^	**14.01** (12.71;15.31)	− **0.33 **(− 0.42; − 024)	− **2.39 **(− 3.96;− 0.82)	0.11 (− 0.02;0.23)	− **0.35 **(− 0.44;− 0.25)	**0.69 **(0.13;1.25)	**2.18 **(1.78;2.59)	0.105
Emotional symptoms^b^	**3.18** (2.71;3.65)	− **0.10 **(− 0.14;− 0.07)	− **0.65 **(− 1.22;− 0.08)	**0.08 **(0.04;0.13)	− **0.07 **(− 0.10;− 0.03)	0.13 (− 0.08;0.33)	0.13 (− 0.02;0.27)	0.028
Conduct problems^b^	**3.16** (2.79;3.52)	− **0.08 **(− 0.11;− 0.06)	− **0.69 **(− 1.13;− 0.24)	0.03 (− 0.01;0.06)	− **0.05 **(− 0.08;− 0.03)	0.14 (− 0.02;0.29)	**0.42 **(0.30;0.53)	0.063
Hyperactivity^b^	**6.40** (5.88;6.92)	− **0.18 **(− 0.22;− 0.15)	− **1.01 **(− 1.64;− 0.39)	0.01 (− 0.04;0.06)	− **0.16 **(− 0.20;− 0.12)	0.19 (− 0.04;0.41)	**0.80 **(0.64;0.96)	0.161
Peer problems^b^	**1.28** (0.87;1.69)	**0.04 **(0.01;0.07)	− 0.05 (− 0.54;0.45)	− 0.01 (− 0.05;0.02)	− **0.07 **(− 0.10;− 0.04)	**0.24 **(0.07;0.42)	**0.84 **(0.71;0.97)	0.079
Generalized anxiety^a^	**4.22** (2.59;5.86)	.00 (− 0.11;0.11)	− 0.64 (− 2.87;1.59)	0.15 (− 0.01;0.30)	− .00 (− 0.08;0.07)	0.11 (− 0.38;0.60)	**0.64 **(0.32;0.95)	0.041

*CI* confidence interval, *HRQoL* health-related quality of life, significant effects are indicated in bold face

^a^Self-reported data (11- to 17-year-olds)

^b^Parent-reported data (7- to 17-year-olds)

With regard to the control variables, older age and female gender were each associated with fewer parent-reported mental health problems in total and on the SDQ subscales, except for peer problems, which increased with age and showed no gender-specific effect. A significant interaction effect indicated that higher age was related to stronger parent-reported emotional problems in girls only. Overall, higher self-reported HRQoL was found in girls, though it decreased with advancing age in this gender group. Higher parental education was associated with fewer parent-reported mental health problems in children. Migration background was related to more total mental health problems and to more severe peer problems (both parent-reported).

### Psychosomatic complaints during the pandemic

Children and adolescents aged 11 to 17 years self-reported substantial psychosomatic complaints; about half of the sample [*n* = 554 (53.2%)] felt irritable and considerable proportions of the sample had sleeping problems [*n* = 449 (43.3%)], headaches [*n* = 421 (40.5%)], felt low [*n* = 352 (33.8%)], and/or reported stomache aches [*n* = 317 (30.5%)]. Girls were affected more than boys with regard to having headaches (*p* = 0.036), stomach aches (*p* = − 0.014) and feeling low (*p* = − 0.002).

### Risk factors for mental health problems during the pandemic

The high-risk group analysis confirmed our hypotheses that children from families with (i) low education levels, or (ii) less than 20 square meters of living space per person, or (iii) a migration background were considered to be at a high risk of suffering a sizable impact due to the COVID-19 pandemic when the family climate, as a resource, was also low (the lowest 20% of all respondents). These high-risk children and adolescents (*n* = 126) reported being substantially burdened by the COVID-19 pandemic significantly more than their peers [42.5% (53.3–31.7) vs. 26.7% (29.4–24.4%), *p* = 0.005] and displayed lower self-reported HRQoL (d-ES = 0.67; *p* < 0.001) and more parent-reported total mental health problems (d-ES = 0.83; *p* < 0.001), emotional symptoms (d-ES = 0.59; *p* < 0.001), conduct problems (d-ES = 0.84; *p* < 0.001), hyperactivity (d-ES = 0.60; *p* < 0.001) and peer problems (d-ES = 0.47; *p* < 0.001) as well as self-reported anxiety (d-ES = 0.37; *p* < 0.001), depressive symptoms (d-ES = 0.64; *p* < 0.001), and psychosomatic complaints (d-ES = 0.67; *p* < 0.001).

## Discussion

To our knowledge, this is the first nationwide representative study on the HRQoL and mental health of children and adolescents during the COVID-19 pandemic. We found that children and adolescents in Germany feel significantly burdened by lockdown, social distancing and homeschooling measures. They experience significantly lower HRQoL and more mental health problems, especially hyperactivity and peer problems. While younger children seem to be more negatively impacted by the pandemic than older children, emotional problems in girls seem to increase by age during the pandemic. Also (particularly young) children may express their stress via psychosomatic complaints, which increased during the pandemic compared to the time before, which is relevant for parents and doctors to take into account when children complain about bodily symptoms. Children and adolescents with low socioeconomic status, low parental education and migrant status are particularly burdened by the effects of the COVID-19 pandemic. Our results are highly relevant to public health and health policy. We suggest careful balancing lockdown/homeschooling measures against the mental health risks of children and strongly call for providing targeted mental health care in communities and kindergardens/schools as prevention and intervention measures to support those outlined children and adolescents being severely stressed by the pandemic.

The results concerning the negative impact of COVID-19 measures on HRQoL and mental health are in line with recent non-representative studies from China, India, Brazil, the US, Spain, Italy, and Germany [17–28]. However, comparing the impacts cross-culturally, children and adolescents in Germany do not seem to be affected as negatively as in other countries such as China, Spain and Italy. Surprisingly, our study did not reveal elevated levels of depression during the COVID-19 lockdown and though a higher level of generalized anxiety was found, the corresponding effect was only negligible compared to pre-pandemic data. In comparison to other countries, German children and adolescents may have been impacted less severely during the initial phase of the pandemic, possibly due to a lower incidence and mortality rate of COVID-19 and softer lockdown measures. A longitudinal study is planned to assess whether depressive and anxiety symptoms in German children and adolescents may increase during the ongoing situation.

Although we did not observe an increase in clinically relevant depressive symptoms, our findings indicate that children and adolescents feel highly burdened, have a significantly higher risk of mental health problems than before the pandemic and suffer from psychosomatic complaints. Our future research will therefore focus on psychosocial resources and resilience factors (e.g., family cohesion and social support) that strengthen the mental health of children and adolescents. Children and adolescents at risk of developing mental health problems need to be identified early on to prevent subclinical mental health problems from developing into manifest mental disorders. Targeted early prevention and intervention services are needed to support young people experiencing mental health problems and their access to health services.

Our study also shows that attention should be paid to children at higher risk of suffering from COVID-19 lockdown consequences, including children with low socioeconomic status (i.e., children from families with low education levels, migration background or limited financial resources), which is in line with research on social inequality and mental health [9]. To reduce the health inequalities identified, nationwide, targeted, and low-threshold preventive measures should be initiated, especially for children from socially deprived backgrounds. Further risk factors for mental health in children found in recent US studies are hardships during the crisis, including caregiving burden, job loss and income loss of the parents [25]. Studies show that parents and children’s mental health and stress are closely intertwined [42, 43] with recent studies performed during the COVID-19 pandemic [44, 45] outlining that several factors lead to a higher stress of parents like being single, parenting young children or children with emotional or behavioral difficulties, having financial hardships or losing childcare. Parents with those risk factors are more likely to develop “burn-out” symptoms during the pandemic and need to be supported to avoid escalations in families including neglect and abuse and also to avoid an increase of parental mental disorders. In times of hardship, it has been shown that mental health deteriorates and aggression increases [46]. Our study indicates a deteriorating family climate, more externalizing behaviour among children and more escalating conflicts at home during the pandemic. Current research and previous health and economic disasters have shown that the risk of child abuse and neglect increases during such times [46, 47] and experts warn that parenting is becoming more violent during the COVID-19 pandemic [48]. Thus, UNICEF, politicians and paediatricians have called for support in maintaining children’s health and welfare [5, 6, 15, 48].

Children and adolescents burdened by the pandemic and potentially at severe mental health risk need to be identified early on to prevent further exacerbation of psychopathology. Along with paediatric researchers, health care professionals and institutions [5, 6, 15, 48], we call for raising awareness of the negative impact this pandemic has on children and adolescents. Society, politicians, educational and health care professionals, as well as parents need to take action to reduce the mental health impact of COVID-19 on children and adolescents. Resources must be allocated and prevention and intervention programs need to be established to support vulnerable children and adolescents and to prepare for a potential second wave of COVID-19 or comparable future events. In addition, we suggest introducing mental health promotion and prevention programs that meet the needs of these children. Guidelines for coping with the pandemic aimed at children and parents, and programs to prevent domestic violence through community-based initiatives have been presented by the United States Centers for Disease Control (CDC) [49]. The European Society for Child and Adolescent Psychiatry (ESCAP) and the American Academy of Child and Adolescent Psychiatry (AACAP) have provided a range of materials for communicating about COVID-19 with children, coping with anxiety and stress, telepsychiatry, and school programs to support children in coping with the pandemic. Moreover, it is recommended that parents talk to their children about the situation and their children’s concerns, listen carefully and create a flexible but consistent daily routine, which can give children stability and security.

The present study has the following limitations: (1) Differences between mental health before and during COVID-19 are attributed to the pandemic. However, a number of other individual and societal factors may have influenced these differences. (2) Due to social distancing measures, this study did not use clinical interviews to assess clinical diagnoses of mental disorders. We did, however, administer internationally recommended, established and validated screening instruments to assess HRQoL and mental health in children. Therefore, our study has the strength to report findings not only from parents but also from the perspective of children themselves. (3) The study results may be affected by response bias such as a social desirability bias or a non-response bias, i.e., this study only included German-speaking children, adolescents and parents with computer literacy and access to digital devices with internet. Thus, findings may not be generalizable to countries other than Germany or to other samples. The participation rate of 45.8% in our study was in the range of child health surveys in the US [50] and the UK [51].

Overall, our findings highlight the significant mental health burden of German children and adolescents during the COVID-19 pandemic. They allow conclusions to be drawn enabling health policy, prevention and clinical practice to provide suitable support in the present crisis and comparable future situations. A planned follow-up study will evaluate how children and adolescents react to the future trajectory of the COVID-19 pandemic, to assess long-term impacts of the pandemic and to investigate resources and resilience factors, which may help children to cope better.

## Supplementary Information

Below is the link to the electronic supplementary material.

Supplementary file1 (DOCX 41 KB)

## Data Availability

The data that support the findings of this study are available from the corresponding author upon reasonable request.
