# Supplementary material for: Impact of the COVID-19 pandemic on quality of life and mental health in children and adolescents in Germany
Source: Eur Child Adolesc Psychiatry. 2021 Jan 25;31(6):879–89. doi: 10.1007/s00787-021-01726-5 (PMC7829493; doi:10.1007/s00787-021-01726-5)
Supplement: Supplementary file 1 — Supplementary file1 (DOCX 41 KB) [file 787_2021_1726_MOESM1_ESM.docx]

**Supplementary Material**

**Manuscript title:** Impact of the COVID-19 pandemic on quality of life and mental health in children and adolescents in Germany

**Journal:** European Child and Adolescent Psychiatry

**Authors:** Ulrike Ravens-Sieberer*, Anne Kaman*, Michael Erhart, Janine Devine, Robert Schlack, Christiane Otto

**Corresponding author:** Prof. Dr. Ulrike Ravens-Sieberer (ravens-sieberer@uke.de)

**Supplementary Table 1.** Sociodemographic characteristics of the COPSY and BELLA subsamples used for the pooled analysis of health-related quality of life in children and adolescents before and during the COVID-19 pandemic.

|  | **COPSY subsample**  **(*n* = 1,016)** | | **BELLA subsample**  **(*n* = 941)** | |
| --- | --- | --- | --- | --- |
|  | ***n* (%)** | ***M (SD)*** | ***n* (%)** | ***M (SD)*** |
| **Age** |  | 14.35 (1.86) |  | 14.35 (1.91) |
| **Gender** |  |  |  |  |
| Male | 500 (49.2) |  | 427 (45.4) |  |
| Female | 516 (50.8) |  | 514 (54.6) |  |
| **Migration background** |  |  |  |  |
| No | 861 (84.7) |  | 824 (87.6) |  |
| Yes | 155 (15.3) |  | 117 (12.4) |  |
| **Parental education** |  |  |  |  |
| Low | 192 (18.9) |  | 58 (6.2) |  |
| Medium | 547 (53.8) |  | 509 (54.1) |  |
| High | 277 (27.3) |  | 374 (39.7) |  |

*Note. M* = mean; *SD* = standard deviation. For the pooled analysis of generalized anxiety, a similar subsample of the BELLA study with comparable sociodemographic characteristics was used (*n* = 1,330). The sociodemographic characteristics are available from the authors upon request.

**Supplementary Table 2.** Sociodemographic characteristics of the COPSY and BELLA subsamples used for the pooled analysis of mental health problems in children and adolescents before and during the COVID-19 pandemic.

|  | **COPSY subsample**  **(*n* = 1,553)** | | **BELLA subsample**  **(*n* = 1,556)** | |
| --- | --- | --- | --- | --- |
|  | ***n* (%)** | ***M (SD)*** | ***n* (%)** | ***M (SD)*** |
| **Age** |  | 12.25 (3.32) |  | 12.51 (2.94) |
| **Gender** |  |  |  |  |
| Male | 779 (50.2) |  | 742 (47.7) |  |
| Female | 774 (49.8) |  | 814 (52.3) |  |
| **Migration background** |  |  |  |  |
| No | 1,309 (84.3) |  | 1,365 (87.7) |  |
| Yes | 244 (15.7) |  | 191 (12.3) |  |
| **Parental education** |  |  |  |  |
| Low | 288 (18.5) |  | 84 (5.4) |  |
| Medium | 882 (56.8) |  | 819 (52.6) |  |
| High | 383 (24.7) |  | 653 (42.0) |  |

*Note. M* = mean; *SD* = standard deviation
